# Proteomic analysis of peripheral blood mononuclear cells isolated from patients with pulmonary tuberculosis: A pilot study from Zanzibar, Tanzania

**DOI:** 10.1371/journal.pone.0281757

**Published:** 2023-02-14

**Authors:** Ahmed Barakat, Even Birkeland, Melissa D. Jørstad, Magalie El Hajj, Msafiri Marijani, Anne Døskeland, Olav Mjaavatten, Frode S. Berven, Tehmina Mustafa

**Affiliations:** 1 Centre for International Health, Department of Global Public Health and Primary Care, University of Bergen, Bergen, Norway; 2 Proteomics Unit at University of Bergen (PROBE), Department of Biomedicine, University of Bergen, Bergen, Norway; 3 Department of Thoracic Medicine, Haukeland University Hospital, Bergen, Norway; 4 Department of Medical Affairs, Partner 4 Health, Paris, France; 5 Department of Diagnostic Services, Mnazi Mmoja Hospital, Zanzibar, The United Republic of Tanzania; The Ohio State University, UNITED STATES

## Abstract

This study aimed at exploring the proteomic profile of PBMCs to predict treatment response in pulmonary tuberculosis (PTB). This was a pilot study conducted among 8 adult patients from Zanzibar, Tanzania with confirmed PTB. Blood samples were collected at baseline, at 2 months of treatment, and at the end of treatment at 6 months. Proteins were extracted from PBMCs and analyzed using LC-MS/MS based label free quantitative proteomics. Overall, 3,530 proteins were quantified across the samples, and 12 differentially expressed proteins were identified at both 2 months of treatment and at treatment completion, which were involved in cellular and metabolic processes, as well as binding and catalytic activity. Seven were downregulated proteins (HSPA1B/HSPA1A, HSPH1, HSP90AA1, lipopolysaccharide-binding protein, complement component 9, calcyclin-binding protein, and protein transport protein Sec31A), and 5 proteins were upregulated (SEC14 domain and spectrin repeat-containing protein 1, leucine-rich repeat-containing 8 VRAC subunit D, homogentisate 1,2-dioxygenase, NEDD8-activating enzyme E1 regulatory subunit, and N-acetylserotonin O-methyltransferase-like protein). The results showed that proteome analysis of PBMCs can be used as a novel technique to identify protein abundance change with anti-tuberculosis treatment. The novel proteins elucidated in this work may provide new insights for understanding PTB pathogenesis, treatment, and prognosis.

## 1. Introduction

Tuberculosis (TB) continues to be a major cause of morbidity and mortality in many low and middle-income countries. Global TB incidence is estimated to be 10.1 million, and mortality 1.3 million [1]. Diagnosis of pulmonary TB (PTB) is based on the detection of *Mycobacterium tuberculosis* (MTB) in the sputum by smear microscopy for acid-fast bacilli (AFB) which is a widely available, simple, and inexpensive tool [2]. The standard treatment for PTB includes 2 months of therapy with isoniazid, rifampicin, pyrazinamide and ethambutol (intensive phase), followed by 4 months of treatment with isoniazid and rifampicin (maintenance phase) [1]. This treatment regimen is considered curative for drug sensitive PTB. Response to TB treatment is monitored by follow-up sputum smear microscopy at 2 and 5 months [3, 4]. Diminishing numbers of AFB to smear-negative status during treatment is considered an indication of treatment success, and the sputum smear conversion is considered as a reliable marker for successful treatment. However, it has been shown in a recent study that viable cultivable bacilli were detected in 6% of patients by culture despite successful sputum smear conversion [5]. These findings highlight the need for improvement in monitoring treatment response by developing a sensitive, specific, and rapid surrogate marker for quantifying TB other than the gold standard of culture. It is also not feasible to perform culture in routine practice for monitoring treatment response in the low-resource high TB-endemic setting due to the need for extensive laboratory resources and the long turn-around time. Furthermore, a large proportion of patients with PTB are sputum-smear negative, and physicians tend to treat patients without bacteriological confirmation if the patients come from well-known endemic areas of TB and have a high suspicion of the disease. Monitoring therapeutic response is thus crucial in these cases to enable timely response if a change in treatment is required [2]. The regression of clinical findings does not always correspond to treatment success, and radiological findings often take longer for complete regression. Although routine biomarkers, such as C-reactive protein [6] and elevated leukocyte count and erythrocyte sedimentation rate, decrease with satisfactory TB response [7, 8], they may not be raised in the chronic milder forms of TB, making them not applicable for treatment monitoring in all forms of TB. There is hence a need for more reliable biomarkers to assess the short and long-term treatment response [1, 9].

Peripheral blood mononuclear cells (PBMCs) are generally used as a model system to investigate immune response in infectious diseases such as TB, where the cell-mediated immune response is mainly responsible for disease processes [10]. Given that PBMCs are the primary cells that play a crucial role in the disease process, their profile is expected to change after containment of MTB by the treatment [11]. The aim of the study was to conduct proteomic profiling of the PBMCs isolated from patients with PTB as a means of exploring the immunopathological changes consequent to TB treatment and the potential of using this information to develop a biomarker for monitoring therapeutic response.

## 2. Materials and methods

### 2.1 Study design and setting

This was a pilot study conducted among adult patients with confirmed PTB who were participating in another larger progressive cohort study on the validation of a new diagnostic test in Zanzibar, Tanzania [12]. Zanzibar is a semi-autonomous region of the United Republic of Tanzania, with a population of around 1.3 million [13]. In recent years, the total number of TB cases has significantly increased in Tanzania from 62,180 cases in 2015 to 75,845 cases in 2018, representing a 22% increase. The notification rate of new and relapse TB cases has also increased from 128 per 100,000 population in 2015 to 138 per 100,000 population in 2018. In contrast, the number of TB cases co-infected with HIV has decreased by more than one-third between 2015 and 2018 [14].

Patients were recruited at Mnazi Mmoja Hospital, which is the only tertiary care referral hospital in Zanzibar, from August 2014 to September 2015 [12]. TB was confirmed bacteriologically by a positive MTB culture and/or MTB detected by the Xpert^®^ MTB/RIF assay (Cepheid Inc, USA) in at least one patient sputum specimen. Patients were excluded if they did not give consent or had received anti-TB treatment in the last 12 months.

Blood samples were collected from each patient at three timepoints: at baseline before starting the treatment "0M", at 2 months (during the intensive phase of treatment) "2M", and at the end of 6 months of treatment "6M".

The study was conducted according to the principles of the Declaration of Helsinki and approved by the Regional Committee for Medical and Health Research Ethics of Western Norway (REK Vest), and the Zanzibar Medical Research and Ethics Committee (ZAMREC). All patients provided written informed consent.

### 2.2 Preparation of proteins from PBMCs and protein digestion

Blood samples (4 mL) were collected using BD Vacutainer^®^ CPT^™^ (cell preparation tubes with sodium heparin). Blood samples were then centrifuged following manufacturer’s instruction, and PBMCs were collected. The PBMCs were subsequently washed thoroughly to remove the plasma, dextran, and other components of the gel in the CPT^™^ tube. The first wash was done with TBS by diluting 1:5. The whole tubes, which were about 6 mL each, were filled up. Then, the samples were centrifuged at 400 × g at room temperature for 10 minutes, to make sure the cells were pelleted. The supernatant was removed. The second wash was done by adding another 4 mL of TBS in the tube with a cell pellet. The pellet was gently dissolved by pipetting in and out a few times. Afterward, another centrifugation at 400 × g at room temperature for 5 minutes was performed to make sure that the cells were pelleted. The supernatant was removed. The third wash was done in a similar manner to the second wash.

Following this, red blood cells were lysed by using a red cell lysis buffer with 150 mM of ammonium chloride. Approximately 2.5 mL of the lysis buffer was added to the cell pellet; then, the tubes were shacked to dissolve the pellet. The tubes were subsequently incubated for 10 minutes at room temperature, and centrifugated at 400 × g at room temperature for 5 minutes. Finally, the supernatant was decanted.

PBMCs pellets were extracted into a 100-μL lysis buffer consisting of 0.1 M Tris/HCl (pH 7.5), 0.1 M dithiothreitol (reducing agent), and 2% SDS. The samples were subsequently transported to the University of Bergen. The proteomics analysis was done at the Proteomics Unit of the University of Bergen (PROBE). Protein concentrations were measured using Direct Detect^®^ (Merck KGaA, Darmstadt, Germany), which is an infrared-based biomolecular quantitation system that provides accurate and precise results despite the presence of SDS. Proteins were digested using the FASP method as described by Hernandez-Valladares et al. [15]. In brief 20 ug of protein were reduced with 0.1 M dithiotreitol (DTT) and heated to 95 °C for 5 min. The proteins were alkylated with 50mM iodoacetamide (IAA). Buffer exchange was performed in a Microcon-30 kDa Centrifugal filters (Millipore, #MRCF0R030) using 8 M urea in 0.1 M Tris–HCl pH 8.5, freshly prepared). Trypsin was dissolved in 50mM ammonium bicarbonate and added to the samples in a 1:25 ratio, samples were incubated at 37 °C for 16 h. Desalting was done using Oasis HLB 96-well μElution plate (2 mg sorbent per well, Waters #186001828BA).

### 2.3 LC/MS method

5 ug of digested peptides were pressure-loaded onto an HPLC column (Acclaim^™^ PepMap^™^ 100 C18, 3 μm, 75 μm × 2 cm, Thermo Fisher Scientific, Bremen, Germany), with trapping and desalting carried out at 5 μL/min for 5 minutes using 0.1% of trifluoroacetic acid. Analytical separation was carried out with Acclaim^™^ PepMap^™^ 100 C18 (3 μm, 75 μm × 50 cm, Thermo Fisher Scientific, Bremen, Germany) at a flow rate of 270 nL/min. The elution gradient was run using mobile phase A (0.1% of formic acid in water) and B (100% ACN). Tryptic peptides underwent a 20-minute isocratic elution with 80% buffer B followed by another 20-minute isocratic elution with 5% buffer B. Total gradient time was 4h. The reason for using a 4h gradient was to increase the number of identified proteins.

As peptides were eluted from the HPLC column, they were electrosprayed directly into a linear quadrupole ion trap-orbitrap mass spectrometer (LTQ-Orbitrap Elite^™^, Thermo Fisher Scientific, Bremen, Germany). The mass spectrometer was operated in the data-dependent acquisition mode to automatically switch between full-scan MS and MS/MS acquisition. Instrument control was through Tune 2.7.0 and Xcalibur 2.2. The mass spectrometric data was acquired in positive ion mode, with an 1,800-V ion spray voltage, no sheath and auxiliary gas flow, and a capillary temperature of 260°C.

Survey full-scan MS spectra (from m/z 300 to 2,000) were acquired in the Orbitrap with a resolution of 240,000 at m/z 400 (after accumulation to a target value of 1e6 in the linear ion trap with the maximum allowed ion accumulation time of 300 ms). The 12 most intense eluting peptides above an ion threshold value of 3,000 counts and charge states of ≥2 were sequentially isolated to a target value of 1e4 and fragmented in the high-pressure linear ion trap by low-energy CID with a normalized collision energy of 35% and wideband-activation enabled. The maximum allowed accumulation time for CID was 150 ms, with an isolation window of 2 Da, an activation q value of 0.25, and an activation time of 10 ms. The resulting fragment ions were scanned out in the low-pressure ion trap at a normal scan rate and recorded with the secondary electron multipliers. One MS/MS spectrum of a precursor mass was allowed before dynamic exclusion for 40 seconds. Lock-mass internal calibration was not enabled.

### 2.4 Data management and analysis

Four softwares were used for this study: MaxQuant v1.5.5.1, Perseus v1.5.6.0, SPSS v25, and Excel 2019.

The raw files from the LC-MS/MS were analyzed using MaxQuant version 1.5.5.1 and the integrated Andromeda search engine. The fasta file version was Sprot_Human_20432entries_20190903.fasta. Moreover, for both proteins and peptides, the maximum FDR was set to 0.01 [16]. MaxQuant maps the sequences of detected peptides and uses these peptide levels to determine the identified protein level. Since the protein levels likely vary between samples due to minor differences in handling and analysis, normalization of protein levels is essential. Consequently, label-free quantification (LFQ) algorithms within MaxQuant, MaxLFQ, were used to create the normalized protein intensities [16]. They were normalized in relation to the levels of common proteins in a sample.

To construct a relative scale, LFQ uses the signal intensity and the number of observations of commonly observed peptides. This is used to assign new, normalized intensities of peptides, along with an absolute scale of summed-up peptide intensities, LFQ intensities. The LFQ algorithm is incorporated into the search engine of MaxQuant and produces two distinctive data outputs: samples without standardized levels and the same samples with LFQ corrected levels. While the unnormalized spectra–"Intensity"–have been merely used to detect the presence of proteins within a sample, the LFQ values–"LFQ intensity"–were used for statistical analysis [17].

The normalized data from MaxQuant were saved in a.txt file [18]. The file was uploaded to Perseus version 1.5.6.0, and "LFQ intensities" were selected as expression data. Potential contaminants, reverse hits, rows only identified by site, and empty rows were removed from the matrix [19]. The different samples were then grouped into "0M", "2M", and "6M", and a matrix was generated. The intensities values were transformed to log2 values, and gene annotations were uploaded for Homo sapiens. S1 Fig in S1 File shows the unsupervised hierarchical clustering of all samples. The variability between samples was very low as shown in S2 File. S1 Table in S2 File provides information about the standard deviation and coefficient of variation of proteins within samples and missing values. S2 Table in S2 File provides information about the standard deviation and coefficient of variation of proteins between samples. S3 Table in S2 File shows the Pearson correlation values between samples. S2 Fig in S1 File illustrates clustering of Pearson correlation values. S3 Fig in S1 File shows the sample distribution of all samples. A histogram was made from each sample were the log2 LFQ intensity was plotted against counts.

To compare the differing expression of proteins detected between the groups, ANOVA was carried out using permutation-based FDR, with the number of randomizations set at 250 which is the default setting in Perseus and the FDR at 0.05. The data were normalized on the protein level with Z-scoring prior to hierarchical clustering. In addition, we performed a mixed linear model analysis with correction for multiple testing for comparison (S4, S5 Figs in S1 File). R-scripts and data output can be found in the S4 Table in S2 File and S1 Data. To visualize the results, a heat map was created to evaluate the significantly differently proteins’ levels between the groups. The data were not imputed.

The significantly expressed proteins of interest were further analyzed with IBM SPSS Statistics 25. Besides one-way ANOVA, the Tukey’s range test was also carried out. After running one-way ANOVA and Tukey’s range test in SPSS, box plots were constructed for each of the significantly expressed proteins to illustrate the mean and the median Log2 intensity differences of these proteins detected in the PBMCs of PTB patients at different treatment time points (0M, 2M and 6M). A Principal component analysis were performed on all samples in Perseus using Benjamini-Hochberg FDR 0.05. The mass spectrometry proteomics data have been deposited to the ProteomeXchange Consortium via the PRIDE partner repository with the dataset identifier PXD029634 [20].

### 2.5 Protein interaction and pathway analysis

The STRING database (STRING v.11.0; www.string-db.org) was used to identify known and predicted functional networks and to predict protein-protein interactions. Gene ontology (GO) annotation (www.geneontology.org) was conducted to classify proteins based on biological process (BP), molecular function (MF) and cellular component (CC) using the Protein Analysis Through Evolutionary Relationships database (www.pantherdb.org). Pathway analysis and biological reactions were performed using Reactome version 72 (www.reactome.org).

## 3. Results

### 3.1 Patient characteristics

Table 1 presents the characteristics of the study participants. Overall, 8 patients with bacteriologically confirmed PTB were enrolled into this pilot study. All patients had a positive sputum smear at 0M. After 2 months of receiving standard TB treatment (isoniazid, rifampicin, pyrazinamide, and ethambutol), 6 patients had negative sputum, while 2 patients continued to have a positive sputum smear, which turned negative at 5 months. At 6M, 6 patients had a negative sputum smear, 2 patients were lost to follow up at the end of the study, and one blood sample at 2M was not analyzed.

**Table 1:** Characteristics of the study participants (n = 8).

Patient characteristic	
Age in years, median (range)	34 (20–52)
Gender, n	
Male	6
Female	2
HIV status, n	
Positive	2
Negative	6
Routine diagnostics, n (positive/total)	
Culture (baseline)	7/7
Sputum smear auramine (baseline)	8/8
Xpert (baseline)	5/5
AFB smear baseline	8/8
AFB smear 2 months	2/8
AFB smear 5 months	0/7*

AFB, acid fast bacilli;

*Sputum smear result at 5 months was missing for one patient. This patient was registered with a negative sputum smear at 2 months.

### 3.2 Proteins changing significantly under and after treatment

In total, 3,697 proteins were quantified across the samples using the LFQ shotgun proteomics analysis in Perseus. After removing potential contaminants, reverse hits, rows only identified by site, and empty rows from the matrix, 3,530 proteins were detected.

**Fig 1:**
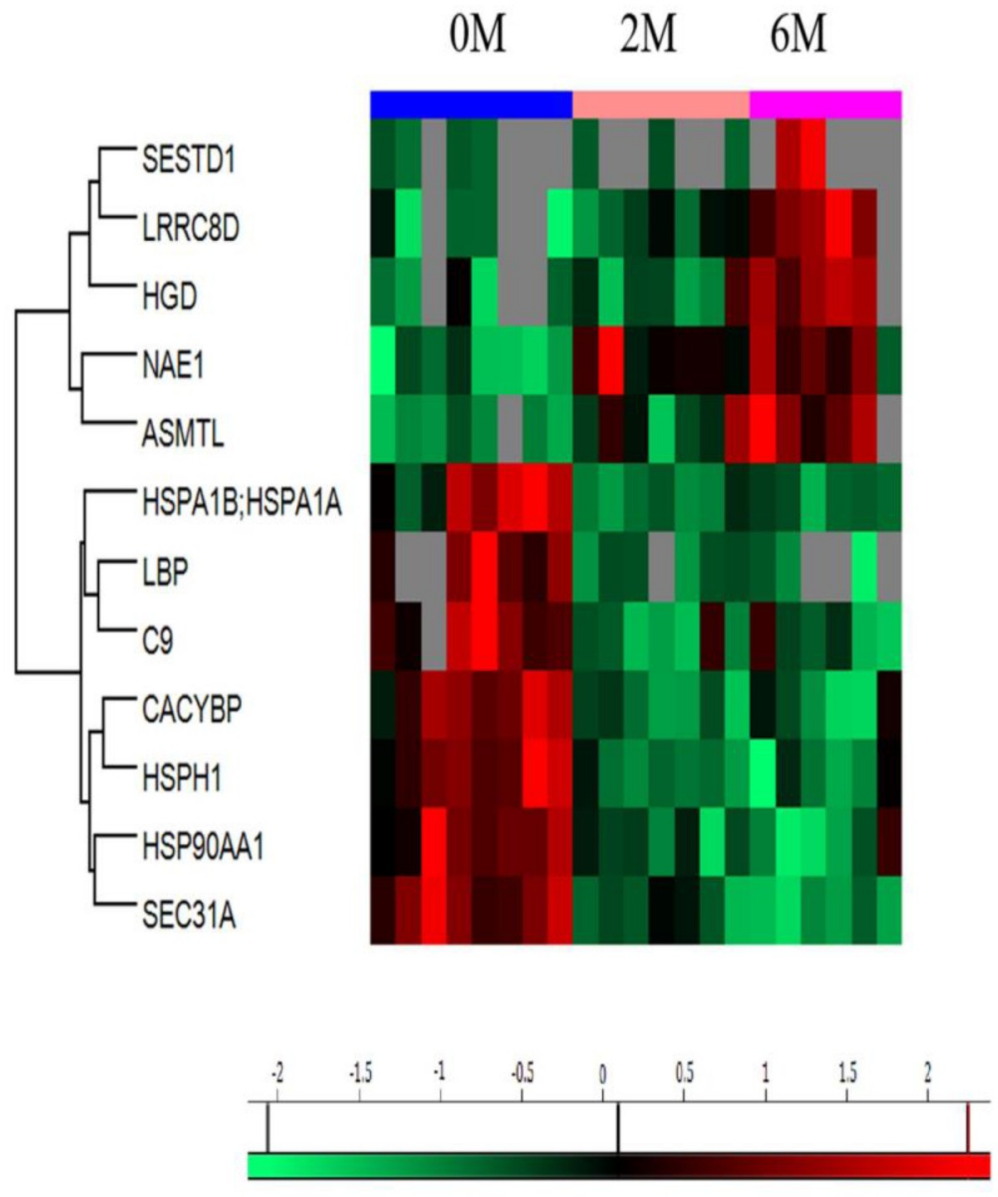
Heat map illustrating hierarchical clusters of the 12 differentially expressed proteins among the pulmonary tuberculosis patients’ PMNCs samples at baseline "0M" (n = 8), 2 months "2M" (n = 7), and 6 months "6M" (n = 6). Each row represents a single protein. The columns depict the expression levels of individual samples. The color intensity of each panel is proportional to the relative intensities of the proteins; higher intensities are associated with red and lower intensities with green.

**Fig 2:**
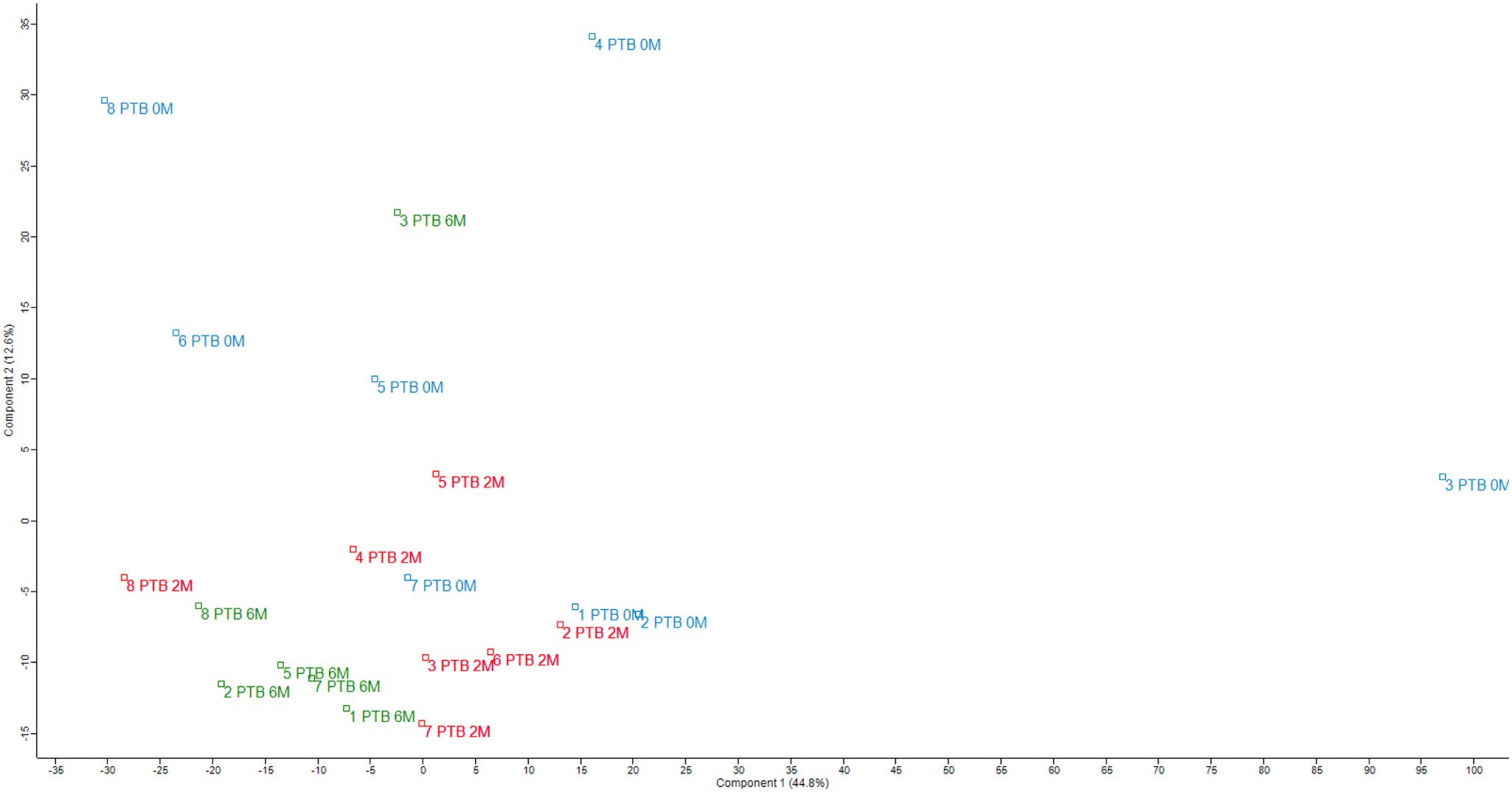
Principal component analysis of all samples. A PCA analysis were performed on all samples in Perseus using Benjamini-Hochberg FDR 0.05.

ANOVA of the log2-transformed LFQ spectra performed on the PTB samples at 0M (n = 8), 2M (n = 7), and 6M (n = 6) detected 12 differentially expressed proteins (Table 2), of which 7 were downregulated, and 5 were upregulated at 2M and 6M. As illustrated in Fig 1, which showcases the hierarchical clustering of the 12 differentially expressed proteins, three main clusters of proteins were identified, and each cluster matched precisely to the treatment progress grouping. Fig 2 shows the principal component analysis of all samples showing the clustering of proteins according to the treatment groups.

**Table 2:** The 12 differentially expressed proteins identified in PBMCs from patients with pulmonary tuberculosis at 2 and 6 months after treatment as compared to the proteins expressed before treatment.

Gene name(s)	Protein name(s)	Log2 fold change	
		0M − 2M	0M − 6M
*LBP*	Lipopolysaccharide binding protein	-5.08	-6.36
*C9*	Complement component C9; Complement component C9a; Complement component C9b	-1.76	-1.58
*HSPA1B; HSPA1A*	Heat shock 70 kDa protein 1B; Heat shock 70 kDa protein 1A	-0.70	-0.67
*HSPH1*	Heat shock protein 105 kDa	-0.68	-0.71
*CACYBP*	Calcyclin-binding protein	-0.55	-0.52
*HSP90AA1*	Heat shock protein HSP 90-alpha	-0.44	-0.54
*SEC31A*	Protein transport protein Sec31A	-0.25	-0.34
*SESTD1*	SEC14 domain and spectrin repeat-containing protein 1	0.03	1
*HGD*	Homogentisate 1,2-dioxygenase	0.21	1.76
*LRRC8D*	Leucine-rich repeat-containing 8 VRAC subunit D	0.30	1.17
*NAE1*	NEDD8-activating enzyme E1 regulatory subunit	0.44	0.48
*ASMTL*	N-acetylserotonin O-methyltransferase-like protein	0.71	1.73

Abbreviations: 0M, baseline; 2M, at 2 months of treatment; 6M, at the end of 6 months of treatment. Anova permutation-based FDR 0.05.

**Fig 3:**
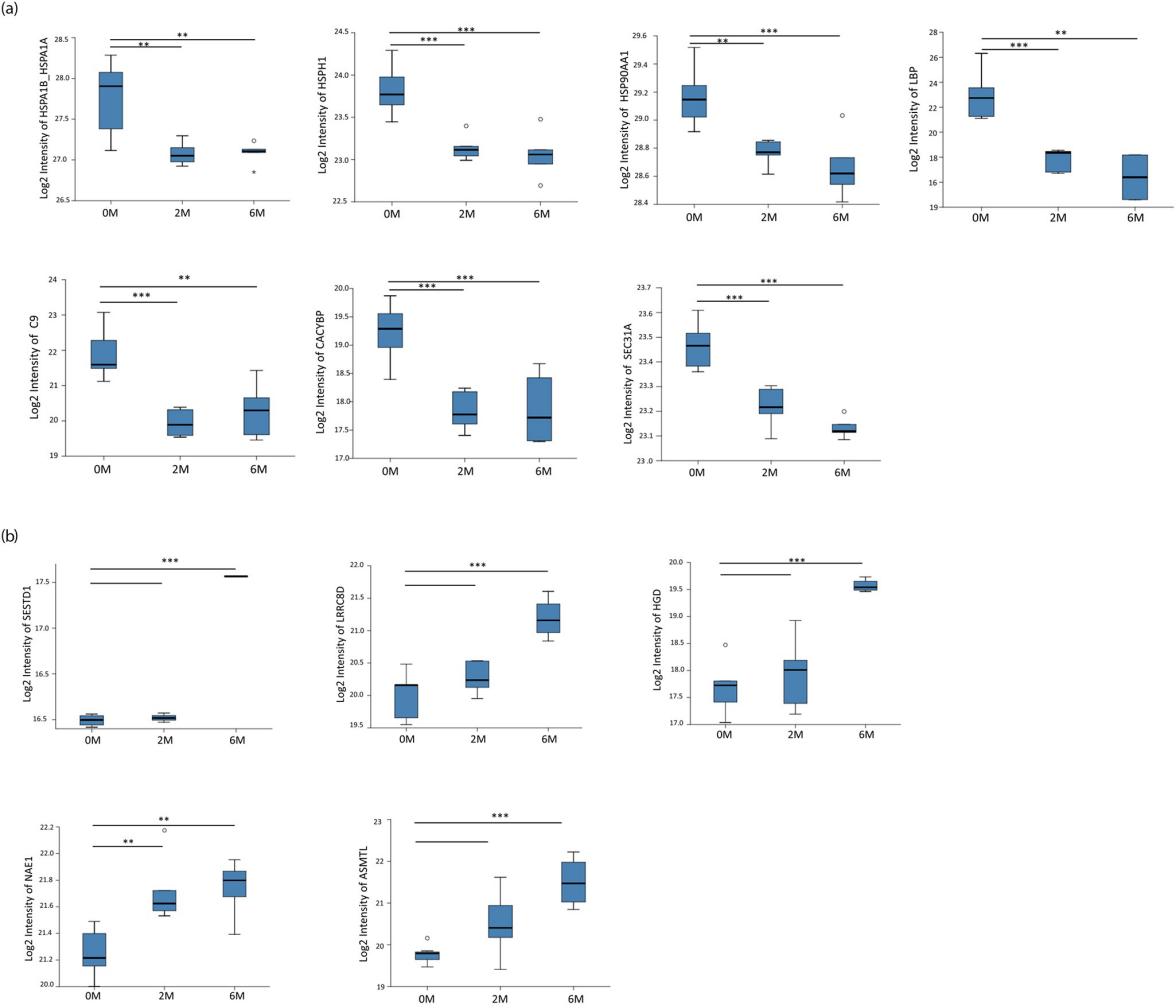
Box plots displaying the median, interquartile range, and the minimum and maximum values of Log2 intensity of the differentially expressed proteins identified among patients with pulmonary tuberculosis. **(A)** represents the downregulated proteins after 6 months of treatment. **(B)** represents the upregulated proteins. A p-value of less than 0.05 indicates statistical significance using ANOVA and Tukey’s range test. *p<0.05; **p<0.01; ***p<0.001. The X-axis represents the time of treatment in months (0M, baseline; 2M, 2 months; 6M, 6 months of treatment). The Y-axis depicts the Log2 Intensity of each of the proteins.

The 7 downregulated proteins identified in the PTB samples at 2M and 6M were heat shock 70 kDa protein 1A/1B (HSPA1B/HSPA1A), heat shock protein 105 kDa (HSPH1), heat shock protein 90 alpha family class A member 1 (HSP90AA1), lipopolysaccharide binding protein (LBP), complement component 9 (C9), calcyclin-binding protein (CACYBP), and protein transport protein Sec31A (SEC31A) (Fig 3A). LBP was reduced to 5- and 6-folds at 2M and 6M respectively, while the fold reduction in the rest of proteins was between 1.76 to 0.25. All these proteins decreased significantly at 2M, and further treatment lead to a relatively lesser decrease. The 5 upregulated proteins were SEC14 domain and spectrin repeat-containing protein 1 (SESTD1), leucine-rich repeat-containing 8 VRAC subunit D (LRRC8D), homogentisate 1,2-dioxygenase (HGD), NEDD8-activating enzyme E1 regulatory subunit (NAE1), and N-acetylserotonin O-methyltransferase-like protein (ASMTL) (Fig 3B).

### 3.3 Functional enrichment and pathway analysis of the proteins changing significantly with treatment

**Fig 4:**
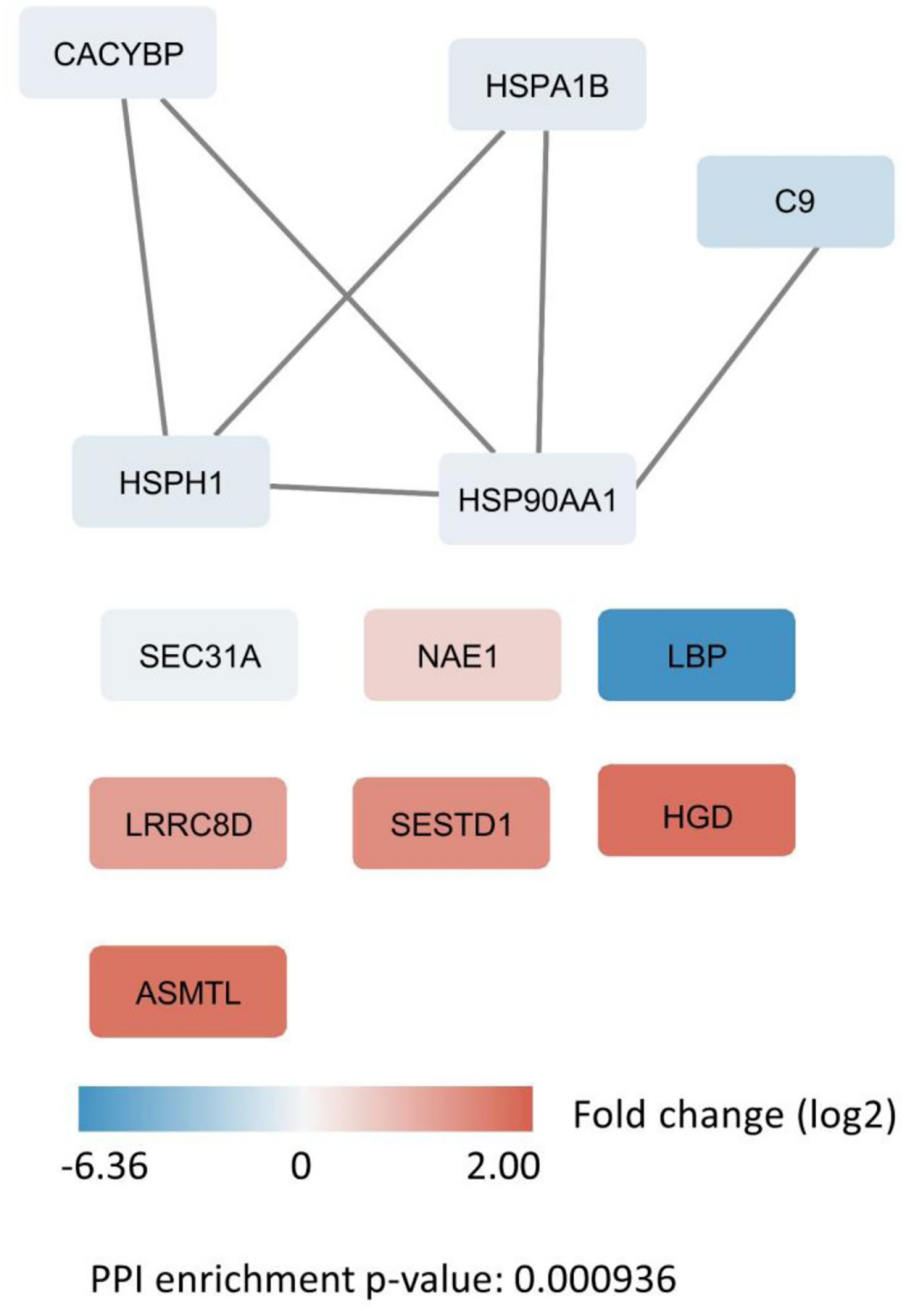
STRING visualization of the protein-protein interaction network for the 12 differentially expressed proteins identified in PBMCs from patients with pulmonary tuberculosis. Proteins are represented squares, where the colors represent fold change. Lines represent known interactions between proteins.

Fig 4 illustrates the protein-protein interaction network analysis consisting of the 12 differentially expressed proteins identified in the PTB samples.

**Fig 5:**
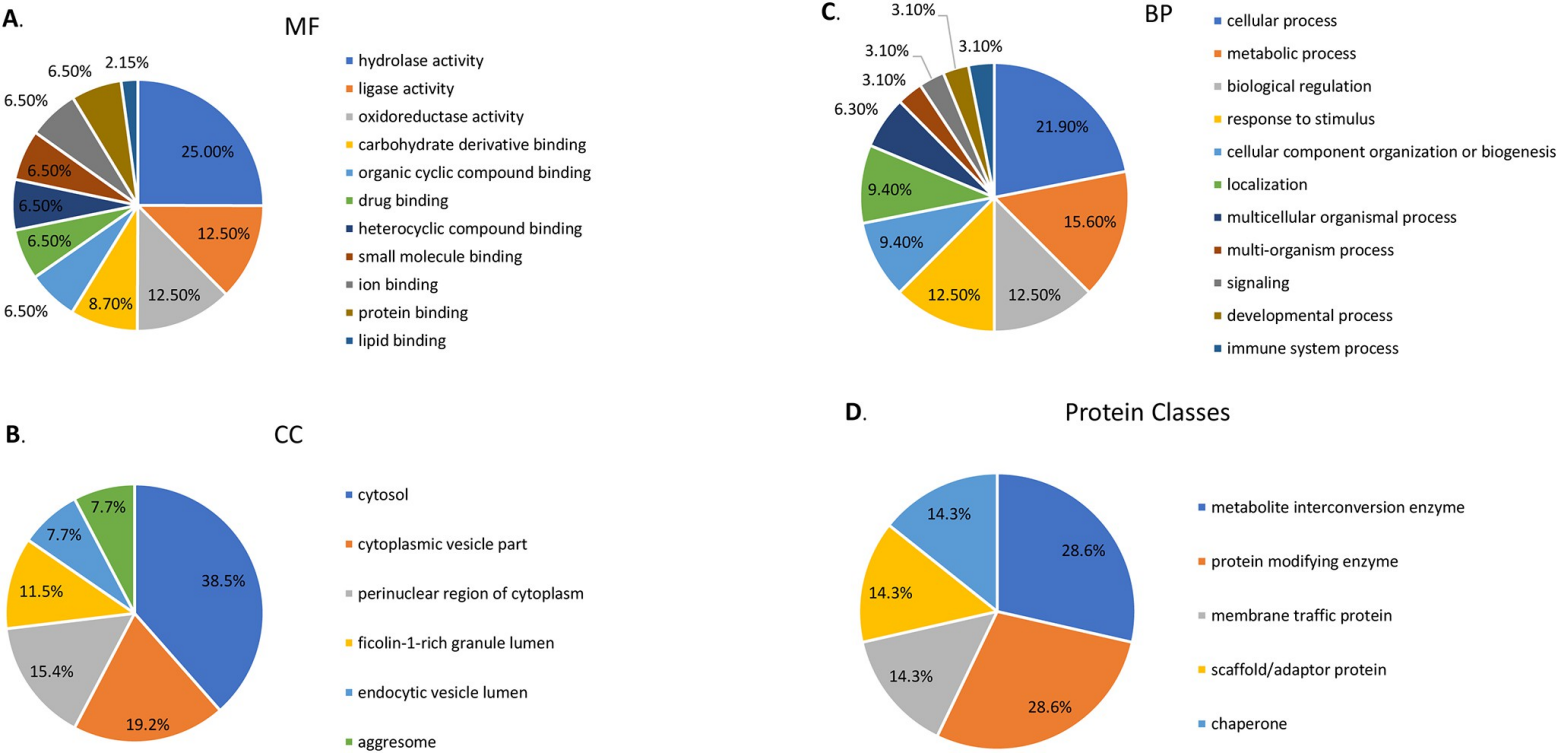
Gene ontology annotations in terms of molecular function (MF) **(A)**, cellular components (CC) **(B)**, biological processes (BP) **(C)**, and protein classes **(D)** for the 12 differentially expressed proteins identified in PBMCs from patients with pulmonary tuberculosis.

Further analysis using GO annotation revealed that the 12 differentially expressed proteins were preferentially associated with MF (Fig 5A), binding (carbohydrate derivative binding, organic cyclic compound binding, drug binding, heterocyclic compound binding, small molecule binding, ion binding, protein binding, lipid binding). As well as activity (hydrolase activity, ligase activity, oxidoreductase activity) were the main affected MFs.

CC proteins (Fig 5B) were mainly associated with the cytosol, the endocytic vesicle lumen, the ficolin-1-rich granule lumen, the aggresome, the perinuclear region of cytoplasm, and the cytoplasmic vesicle part.

In terms of BP (Fig 5C) the proteins were involved process (cellular process, metabolic process, multicellular organismal process, multi-organism process, developmental process, immune system process), biological regulation, response to stimulus, cellular component organization or biogenesis, and localization.

Regarding protein classes (Fig 5D), the main classes of identified proteins were metabolite interconversion enzymes (HGD and ASMTL), protein modifying enzymes (NAE1 and CACYBP), membrane traffic proteins (SEC31A), scaffold/adaptor proteins (LRRC8D), and chaperone proteins (HSP90AA1).

A reactome pathway analysis found that the following pathways were highly represented: scavenging by class F receptors, cellular response to heat stress, attenuation phase, HSP90 chaperone cycle for steroid hormone receptors, regulation of heat shock factor 1-mediated heat shock response, interleukin-4 and interleukin-13 signaling, and innate immune response.

## 4. Discussion

In this study, we have shown for the first time the exploration of the proteome of PBMCs for biomarker discovery to predict response to treatment in TB. In total, 3,530 proteins were quantified across the samples, and 12 differentially expressed proteins were identified in patients with PTB. Based on our results, we speculate that the testing of these 12 proteins by using routine laboratory assays early during treatment will ensure the timely management of patients not responding to anti-TB treatment. This is of great value where anti-TB treatment is started without bacteriological confirmation [12].

LBP is a 60-kDa serum glycoprotein, which plays a role in the innate immune response and antibacterial defense through the activation of neutrophil-producing reactive oxygen species that can kill bacteria [21]. In a small 2013 study conducted among 36 children with TB, LBP was found to be a marker of innate immune system activation [22]. In line with our findings, a study from Uganda using serum proteomics in 39 patients with PTB identified LBP as an important serum biomarker associated with PTB treatment response, as its concentration significantly decreased between baseline and 2 months of therapy [23]. The 5-fold decrease in the levels of LBP after treatment that was found in the present study makes it a suitable candidate for further investigation as a potential biomarker for therapeutic monitoring as early as 2 months after treatment.

C9 is a part of a complementary membrane attack complex/perforin domain, and is also a marker of innate immune system activation [24]. In a recently published quantitative proteomics study aiming to identify specific protein signatures in sera of active TB patients and their household contacts, C9 was found to be highly accumulated in the serum of active TB patients [25]. However, a significant change in the levels of this protein with treatment has not been shown in earlier studies. The 1.76-fold decrease in its levels after 2 months of treatment implies its role in monitoring therapeutic response.

HSPs are numerous cell proteins involved in the homeostasis of proteins [26]. In TB infection, HSPs exhibit different functions, including the activation of toll-like receptors which in turn activates pro-inflammatory signals, eliciting immune responses [26]. These proteins have been evaluated as a tool for TB diagnosis, and a potent vaccine candidates [27, 28]. However, to the best of our knowledge, no study has evaluated HSPs as markers of treatment response in patients with TB.

Besides LBP, C9, HSP70, and HSP90, the other differentially expressed proteins have not been previously reported to be associated with either TB diagnosis or treatment response. Thus, our novel data contribute to a further understanding of the complexity of changes accompanying TB treatment. The proteins increasing in response to treatment imply their role in the protective immune response against TB.

Other protein biomarkers, including soluble intercellular adhesion molecule 1, soluble urokinase plasminogen activator receptor, and procalcitonin have demonstrated significant decrease in levels following treatment of PTB [29, 30]. Several studies using whole blood transcriptome analysis have shown significant changes in response to receiving TB treatment; In 2012, 320-transcript signature were significantly diminished in response to treatment [31]. Another study in 2017 had noticed 5-gene signature correlated to TB treatment [32]. The present study did not identify any of these biomarkers. This could be due to the difference in the study of RNAs in the transcriptome studies and proteins in our study, and all RNAs may not be translated into the proteins. Furthermore, only PBMCs were used in our study, as many relevant proteins would be present only in the plasma. Thus, the best approach would be to combine the PBMCs and the plasma proteomics for a comprehensive biomarker discovery for TB treatment monitoring with an increased specificity and high predictive value [33].

Interestingly, our data highlighted an enrichment of GO terms related to cellular and metabolic processes, as well as binding and catalytic activity. This is consistent with the results of several recent quantitative proteomics studies from China conducted among TB patients with or without HIV [34, 35]. This demonstrates that differentially expressed proteins identified in PBMCs from patients with TB have multiple biological functions that require further investigation.

Our study is strengthened by the use of an HPLC column, which is associated with minimal disruption of the native condition of the samples, simple procedure, reproducible results, and high capacity [36]. A second methodological strength in the present study was the use of MS with Orbitrap Elite^™^, characterized by a high resolution as well as high scan speeds [17]. More importantly, the major strength of this study is the detailed mapping of the PBMC proteome. Most proteomic TB studies have focused mainly on serum or plasma as the primary source of sampling. However, using PBMC samples instead of plasma samples for proteomic profiling has several advantages. First, PBMCs can be obtained relatively easily from routinely collected blood samples and thus provide direct access to physiologically important immune proteins without the well-known analytical complexities of the presence of highly abundant proteins in native human plasma [19]. Second, in contrast to proteomic analyses of plasma samples, proteomic profiling of PBMCs can detect low-abundant proteins from blood which can represent valuable biomarker candidates [37]. Third, PBMCs have been found to be significantly richer as a source of biomarkers compared to plasma [37]. In an experimental study comparing the PBMC proteome to the plasma proteome obtained from blood as the same source sample, the number of proteins identified in PBMCs as a cellular compartment of blood (4,129 proteins) was more than double the amount of proteins reported in plasma (1,929 proteins) [37]. Hence, PBMCs as a blood-derived cellular sample represents a valuable sample for TB biomarker studies, and both PBMC samples and plasma samples should be used for a comprehensive proteomic analysis, as these two sample types have been found to encode different proteins [37].

Despite the novelty of our findings, there are several limitations to our study, including a small sample size, and the omnipresence of pre-analytical variability. The lack of suitable controls such as non-responders to treatment make it difficult to distinguish if the differentially expressed proteins represent the host response to the anti-tuberculosis drugs and the toxicity of this treatment rather than the specific response to treatment. Nevertheless, our findings provide a platform for future investigation into the use of biomarkers from PBMCs to assess treatment efficacy in TB. Due to the high number of biomarker candidates identified in the discovery phase by unbiased proteomics, and the costs of assay development and validation, a prioritized selection of the differentially expressed proteins should be performed in future studies using ELISA or western blot based on the fold-change between baseline and post-treatment (as the proteins with the highest fold-change might be the most attractive biomarkers) and relation with TB pathogenesis.

In conclusion, proteome analysis of PBMCs can be used as a novel technique to identify potential biomarkers to assess treatment efficacy in patients with PTB. Overall, 3,530 proteins were identified based on LC-MS/MS-based label-free quantitative analysis, and a total of 12 proteins were found to be significantly affected by PTB treatment. The novel proteins elucidated in this work may provide new insights for understanding TB pathogenesis, treatment, and prognosis. Further studies are however needed with a larger sample size and controls to validate our results.

## Supporting information

S1 File (PPTX)

S2 File (XLSX)

S1 Data Supplementary R script. (R)

## Abbreviations

AFB: acid-fast bacillus
ASMTL: N-acetylserotonin O-methyltransferase-like protein
BP: biological process
C9: complement component 9
CACYBP: calcyclin-binding protein
CC: cellular component
GO: gene ontology
HGD: homogentisate 1,2-dioxygenase
LFQ: label-free quantification
LRRC8D: leucine-rich repeat-containing 8 VRAC subunit D
MF: molecular function
MTB: *Mycobacterium tuberculosis*
NAE1: NEDD8-activating enzyme E1 regulatory subunit
PROBE: Proteomics Unit of the University of Bergen
PTB: pulmonary tuberculosis
SEC31A: protein transport protein Sec31A
SESTD1: SEC14 domain and spectrin repeat-containing protein 1
TB: tuberculosis

## References

[1] WHO. Global tuberculosis report 2019. 2019. https://apps.who.int/iris/bitstream/handle/10665/329368/9789241565714-eng.pdf?ua=1

[2] WHO. Companion Handbook to the WHO Guidelines for the Programmatic Management of Drug-Resistant Tuberculosis. Geneva: World Health Organization; 2014. https://www.ncbi.nlm.nih.gov/books/NBK247422/. 25320836

[3] WHO. Treatment of tuberculosis: guidelines– 4th ed. 2010 Aug. https://apps.who.int/iris/bitstream/handle/10665/44165/9789241547833_eng.pdf?sequence=1. 23741786

[4] Harries AD, Gausi F, Salaniponi FM. When are follow-up sputum smears actually examined in patients treated for new smear-positive pulmonary tuberculosis? Int J Tuberc Lung Dis. 2004;8: 440–444. 15141736

[5] Ambreen A, Jamil M, Rahman MAU, Mustafa T. Viable Mycobacterium tuberculosis in sputum after pulmonary tuberculosis cure. BMC Infect Dis. 2019;19: 923. doi: 10.1186/s12879-019-4561-7 31666021 PMC6822412

[6] Wilson D, Moosa MYS, Cohen T, Cudahy P, Aldous C, Maartens G. Evaluation of tuberculosis treatment response with serial C-reactive protein measurements. Open Forum Infect Dis. 2018;5: 1–8. doi: 10.1093/ofid/ofy253 30474046 PMC6240901

[7] Martins C, Gama AC de C, Valcarenghi D, Batschauer AP de B. Markers of acute-phase response in the treatment of pulmonary tuberculosis. J Bras Patol e Med Lab. 2014;50: 428–433,. doi: 10.5935/1676-2444.20140052

[8] Veenstra H, Baumann R, Carroll NM, Lukey PT, Kidd M, Beyers N, et al. Changes in leucocyte and lymphocyte subsets during tuberculosis treatment; prominence of CD3dimCD56+ natural killer T cells in fast treatment responders. Clin Exp Immunol. 2006;145: 252–260. doi: 10.1111/j.1365-2249.2006.03144.x 16879244 PMC1809688

[9] Wilby KJ, Ensom MHH, Marra F. Review of evidence for measuring drug concentrations of first-line antitubercular agents in adults. Clin Pharmacokinet. 2014;53: 873–90. doi: 10.1007/s40262-014-0170-1 25172553

[10] Iikura K, Katsunuma T, Saika S, Saito S, Ichinohe S, Ida H, et al. Peripheral Blood Mononuclear Cells from Patients with Bronchial Asthma Show Impaired Innate Immune Responses to Rhinovirus in vitro. Int Arch Allergy Immunol. 2011;155: 27–33. doi: 10.1159/000327262 21646792

[11] Kundu J, Bakshi S, Joshi H, Bhadada SK, Verma I, Sharma S. Proteomic profiling of peripheral blood mononuclear cells isolated from patients with tuberculosis and diabetes copathogenesis: A pilot study. bioRxiv. 2020. doi: 10.1371/journal.pone.0233326 33156824 PMC7647457

[12] Jørstad MD, Marijani M, Dyrhol-Riise AM, Sviland L, Mustafa T. MPT64 antigen detection test improves routine diagnosis of extrapulmonary tuberculosis in a low-resource setting: A study from the tertiary care hospital in Zanzibar. Santin M, editor. PLoS One. 2018;13. doi: 10.1371/journal.pone.0196723 29742144 PMC5942825

[13] National Bureau of Statistics (NBS) and Office of Chief Government Statistician (OCGS) Zanzibar. The 2012 Population and Housing Census: Population Distribution by Age and Sex. Dar es Salaam, United Republic of Tanzania: NBS and OCGS. 2013. 2013. http://tanzania.countrystat.org/fileadmin/user_upload/countrystat_fenix/congo/docs/Population Distribution by Age and Sex Report-2012PHC.pdf.

[14] National Tuberculosis and Leplosy Program. TB Prevalence in Tanzania. https://ntlp.go.tz/tuberculosis/tb-prevalence/

[15] Hernandez-Valladares M, Aasebø E, Mjaavatten O, Vaudel M, Bruserud Ø, Berven F, et al. Reliable FASP-based procedures for optimal quantitative proteomic and phosphoproteomic analysis on samples from acute myeloid leukemia patients. Biol Proced Online. 2016;18. doi: 10.1186/s12575-016-0043-0 27330413 PMC4915068

[16] Cox J, Neuhauser N, Michalski A, Scheltema RA, Olsen J V., Mann M. Andromeda: A Peptide Search Engine Integrated into the MaxQuant Environment. J Proteome Res. 2011;10. doi: 10.1021/pr101065j 21254760

[17] Michalski A, Damoc E, Lange O, Denisov E, Nolting D, Müller M, et al. Ultra High Resolution Linear Ion Trap Orbitrap Mass Spectrometer (Orbitrap Elite) Facilitates Top Down LC MS/MS and Versatile Peptide Fragmentation Modes. Mol Cell Proteomics. 2012;11. doi: 10.1074/mcp.O111.013698 22159718 PMC3316736

[18] Cox J, Hein MY, Luber CA, Paron I, Nagaraj N, Mann M. Accurate Proteome-wide Label-free Quantification by Delayed Normalization and Maximal Peptide Ratio Extraction, Termed MaxLFQ. Mol Cell Proteomics. 2014;13: 2513–2526. doi: 10.1074/mcp.M113.031591 24942700 PMC4159666

[19] Anderson NL, Anderson NG. The Human Plasma Proteome. Mol Cell Proteomics. 2002;1: 845–867. doi: 10.1074/mcp.R200007-MCP200 12488461

[20] Perez-Riverol Y, Csordas A, Bai J, Bernal-Llinares M, Hewapathirana S, Kundu DJ, et al. The PRIDE database and related tools and resources in 2019: improving support for quantification data. Nucleic Acids Res. 2019;47: D442–D450. doi: 10.1093/nar/gky1106 30395289 PMC6323896

[21] von Schlieffen E, Oskolkova O V., Schabbauer G, Gruber F, Blüml S, Genest M, et al. Multi-Hit Inhibition of Circulating and Cell-Associated Components of the Toll-Like Receptor 4 Pathway by Oxidized Phospholipids. Arterioscler Thromb Vasc Biol. 2009;29: 356–362. doi: 10.1161/ATVBAHA.108.173799 19112167

[22] Pavan Kumar N, Anuradha R, Andrade BB, Suresh N, Ganesh R, Shankar J, et al. Circulating Biomarkers of Pulmonary and Extrapulmonary Tuberculosis in Children. Clin Vaccine Immunol. 2013;20: 704–711. doi: 10.1128/CVI.00038-13 23486418 PMC3647760

[23] De Groote MA, Nahid P, Jarlsberg L, Johnson JL, Weiner M, Muzanyi G, et al. Elucidating Novel Serum Biomarkers Associated with Pulmonary Tuberculosis Treatment. Sim RB, editor. PLoS One. 2013;8. doi: 10.1371/journal.pone.0061002 23637781 PMC3630118

[24] Spicer BA, Law RHP, Caradoc-Davies TT, Ekkel SM, Bayly-Jones C, Pang S-S, et al. The first transmembrane region of complement component-9 acts as a brake on its self-assembly. Nat Commun. 2018;9: 3266. doi: 10.1038/s41467-018-05717-0 30111885 PMC6093860

[25] Mateos J, Estévez O, González-Fernández Á, Anibarro L, Pallarés Á, Reljic R, et al. Serum proteomics of active tuberculosis patients and contacts reveals unique processes activated during Mycobacterium tuberculosis infection. Sci Rep. 2020;10: 3844. doi: 10.1038/s41598-020-60753-5 32123229 PMC7052228

[26] Bolhassani A, Agi E. Heat shock proteins in infection. Clin Chim Acta. 2019;498: 90–100. doi: 10.1016/j.cca.2019.08.015 31437446

[27] Shekhawat SD, Purohit HJ, Taori GM, Daginawala HF, Kashyap RS. Evaluation of heat shock proteins for discriminating between latent tuberculosis infection and active tuberculosis: A preliminary report. J Infect Public Health. 2016;9: 143–152. doi: 10.1016/j.jiph.2015.07.003 26300163

[28] Bulut Y, Michelsen KS, Hayrapetian L, Naiki Y, Spallek R, Singh M, et al. Mycobacterium Tuberculosis Heat Shock Proteins Use Diverse Toll-like Receptor Pathways to Activate Pro-inflammatory Signals. J Biol Chem. 2005. doi: 10.1074/jbc.M411379200 15809303

[29] Wallis RS, Pai M, Menzies D, Doherty TM, Walzl G, Perkins MD, et al. Biomarkers and diagnostics for tuberculosis: progress, needs, and translation into practice. Lancet. 2010;375: 1920–1937. doi: 10.1016/S0140-6736(10)60359-5 20488517

[30] Gaikwad UN, Gaikwad NR. Modalities to monitor the treatment response in tuberculosis. Indian J Tuberc. 2018;65: 109–117. doi: 10.1016/j.ijtb.2017.12.014 29579423

[31] Bloom CI, Graham CM, Berry MPR, Wilkinson KA, Oni T, Rozakeas F, et al. Detectable Changes in The Blood Transcriptome Are Present after Two Weeks of Antituberculosis Therapy. Doherty TM, editor. PLoS One. 2012;7. doi: 10.1371/journal.pone.0046191 23056259 PMC3462772

[32] Thompson EG, Du Y, Malherbe ST, Shankar S, Braun J, Valvo J, et al. Host blood RNA signatures predict the outcome of tuberculosis treatment. Tuberculosis. 2017; 48–58. doi: 10.1016/j.tube.2017.08.004 29050771 PMC5658513

[33] Pollock NR, Macovei L, Kanunfre K, Dhiman R, Restrepo BI, Zarate I, et al. Validation of Mycobacterium tuberculosis Rv1681 Protein as a Diagnostic Marker of Active Pulmonary Tuberculosis. J Clin Microbiol. 2013;51: 1367–1373. doi: 10.1128/JCM.03192-12 23390284 PMC3647908

[34] Liu Q, Pan L, Han F, Luo B, Jia H, Xing A, et al. Proteomic profiling for plasma biomarkers of tuberculosis progression. Mol Med Rep. 2018;18: 1551–1559. doi: 10.3892/mmr.2018.9134 29901122 PMC6072192

[35] Jiang T-T, Shi L-Y, Wei L-L, Li X, Yang S, Wang C, et al. Serum amyloid A, protein Z, and C4b-binding protein β chain as new potential biomarkers for pulmonary tuberculosis. Kumar S, editor. PLoS One. 2017;12. doi: 10.1371/journal.pone.0173304 28278182 PMC5344400

[36] de Roos B, Duthie SJ, Polley ACJ, Mulholland F, Bouwman FG, Heim C, et al. Proteomic Methodological Recommendations for Studies Involving Human Plasma, Platelets, and Peripheral Blood Mononuclear Cells. J Proteome Res. 2008;7. doi: 10.1021/pr700714x 18489134

[37] Končarević S, Lößner C, Kuhn K, Prinz T, Pike I, Zucht H-D. In-Depth Profiling of the Peripheral Blood Mononuclear Cells Proteome for Clinical Blood Proteomics. Int J Proteomics. 2014. doi: 10.1155/2014/129259 24724028 PMC3958665

