# Supplementary material for: Proteomic analysis of peripheral blood mononuclear cells isolated from patients with pulmonary tuberculosis: A pilot study from Zanzibar, Tanzania
Source: PLoS One. 2023 Feb 14;18(2):e0281757. doi: 10.1371/journal.pone.0281757 (PMC9928017; doi:10.1371/journal.pone.0281757)
Supplement: S1 File — (PPTX) [file pone.0281757.s001.pptx]

## Slide 1
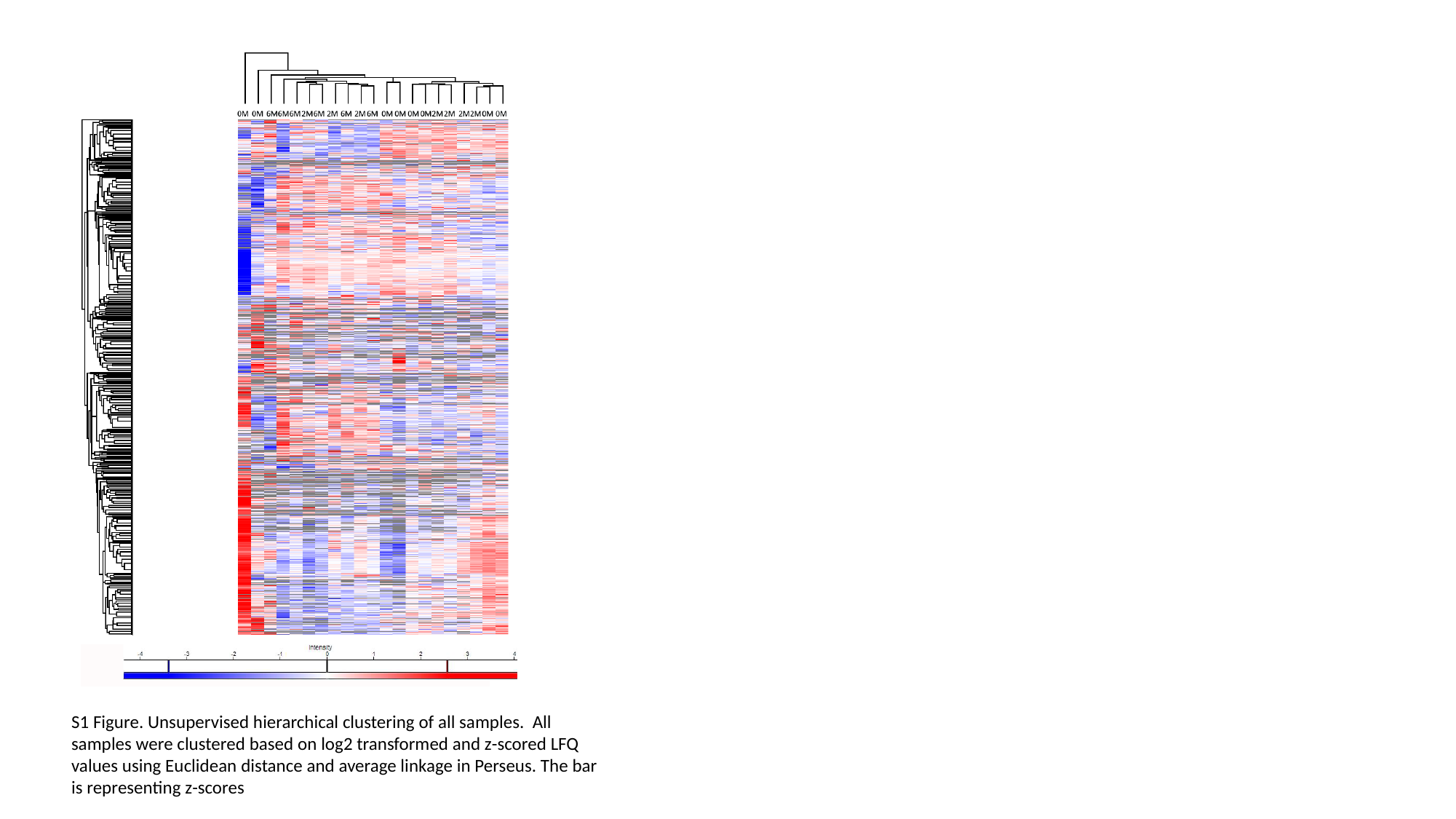

S1 Figure. Unsupervised hierarchical clustering of all samples. All samples were clustered based on log2 transformed and z-scored LFQ values using Euclidean distance and average linkage in Perseus. The bar is representing z-scores

## Slide 2
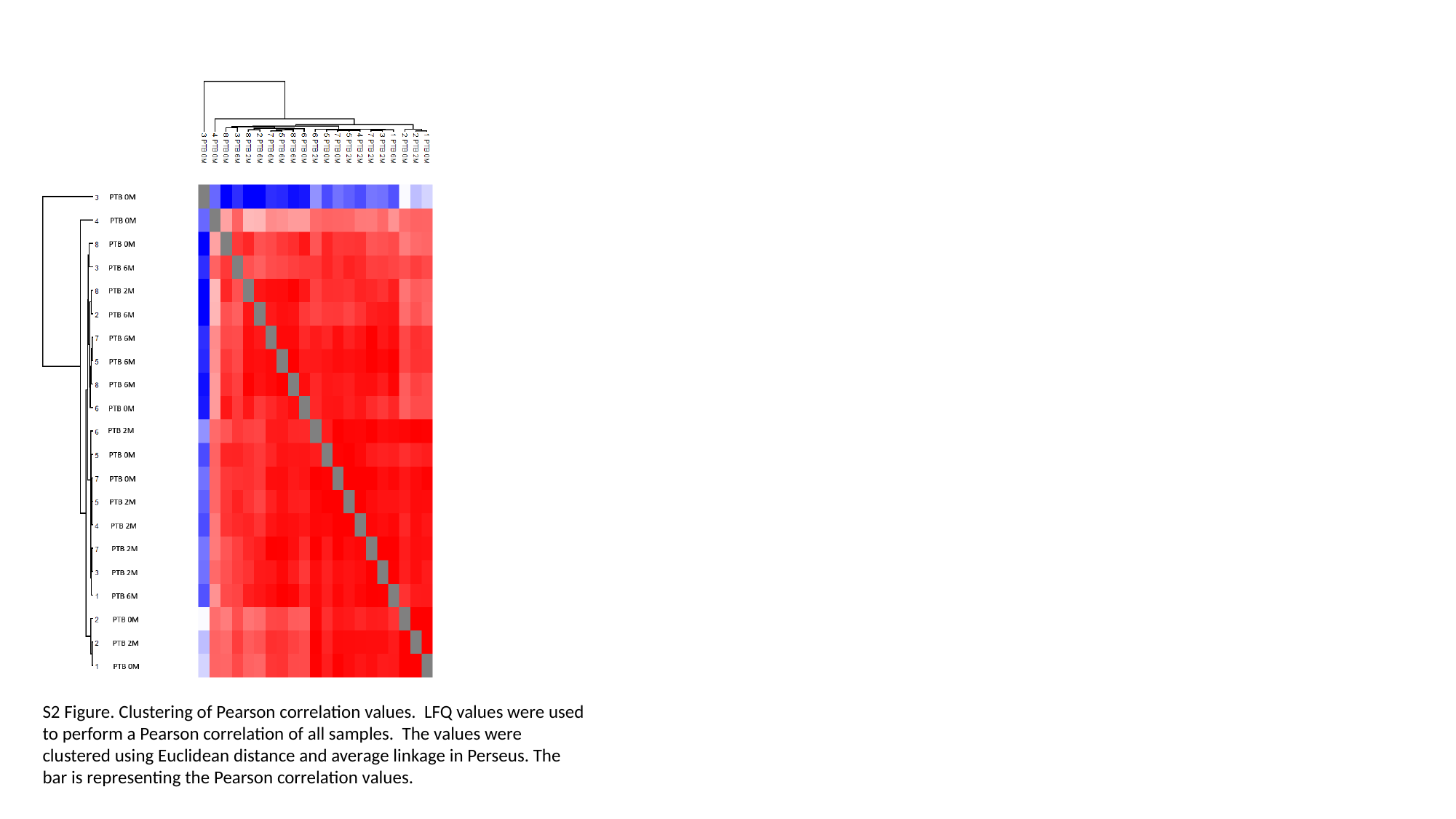

S2 Figure. Clustering of Pearson correlation values. LFQ values were used to perform a Pearson correlation of all samples. The values were clustered using Euclidean distance and average linkage in Perseus. The bar is representing the Pearson correlation values.

## Slide 3
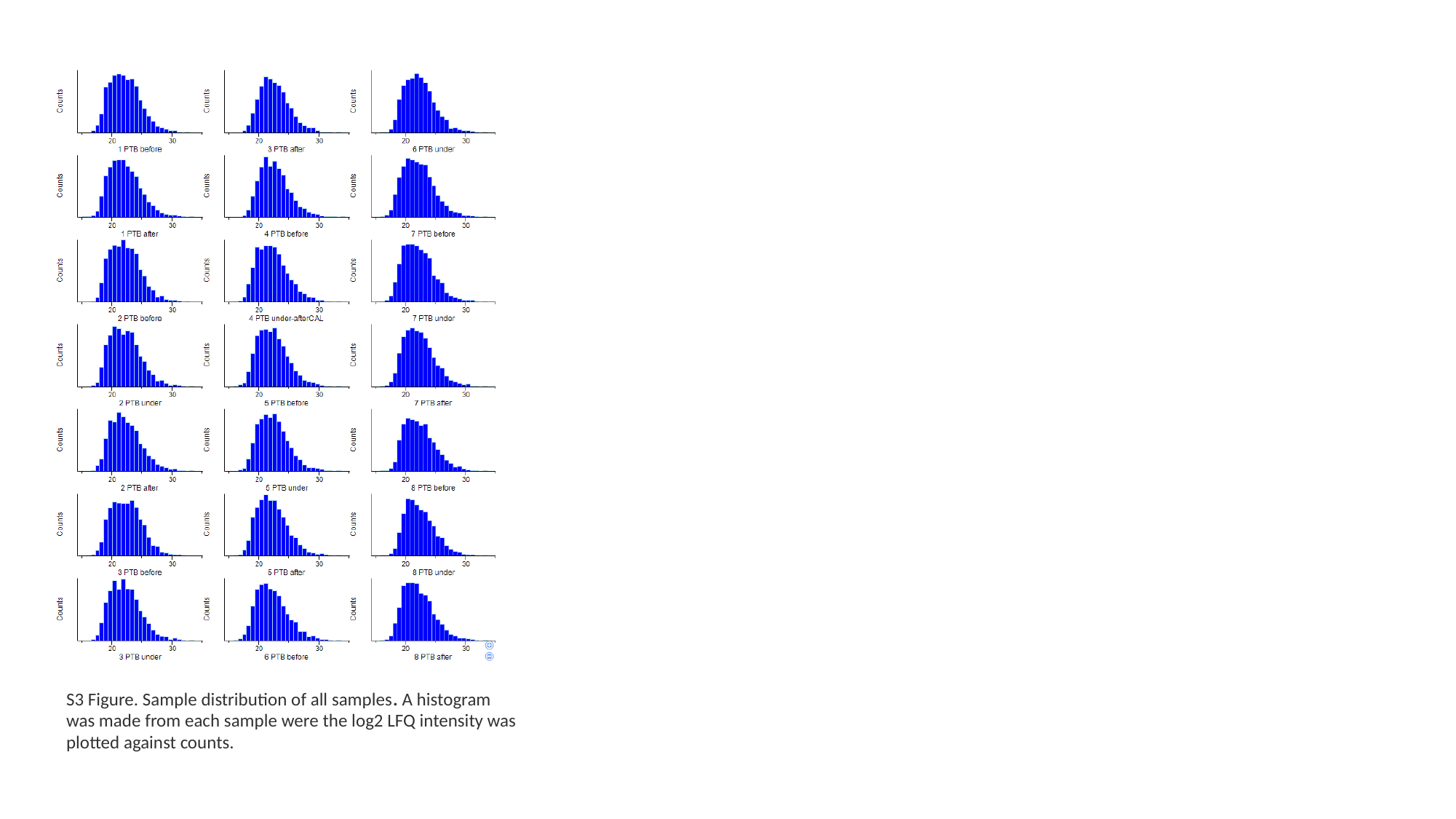

S3 Figure. Sample distribution of all samples. A histogram was made from each sample were the log2 LFQ intensity was plotted against counts.

## Slide 4
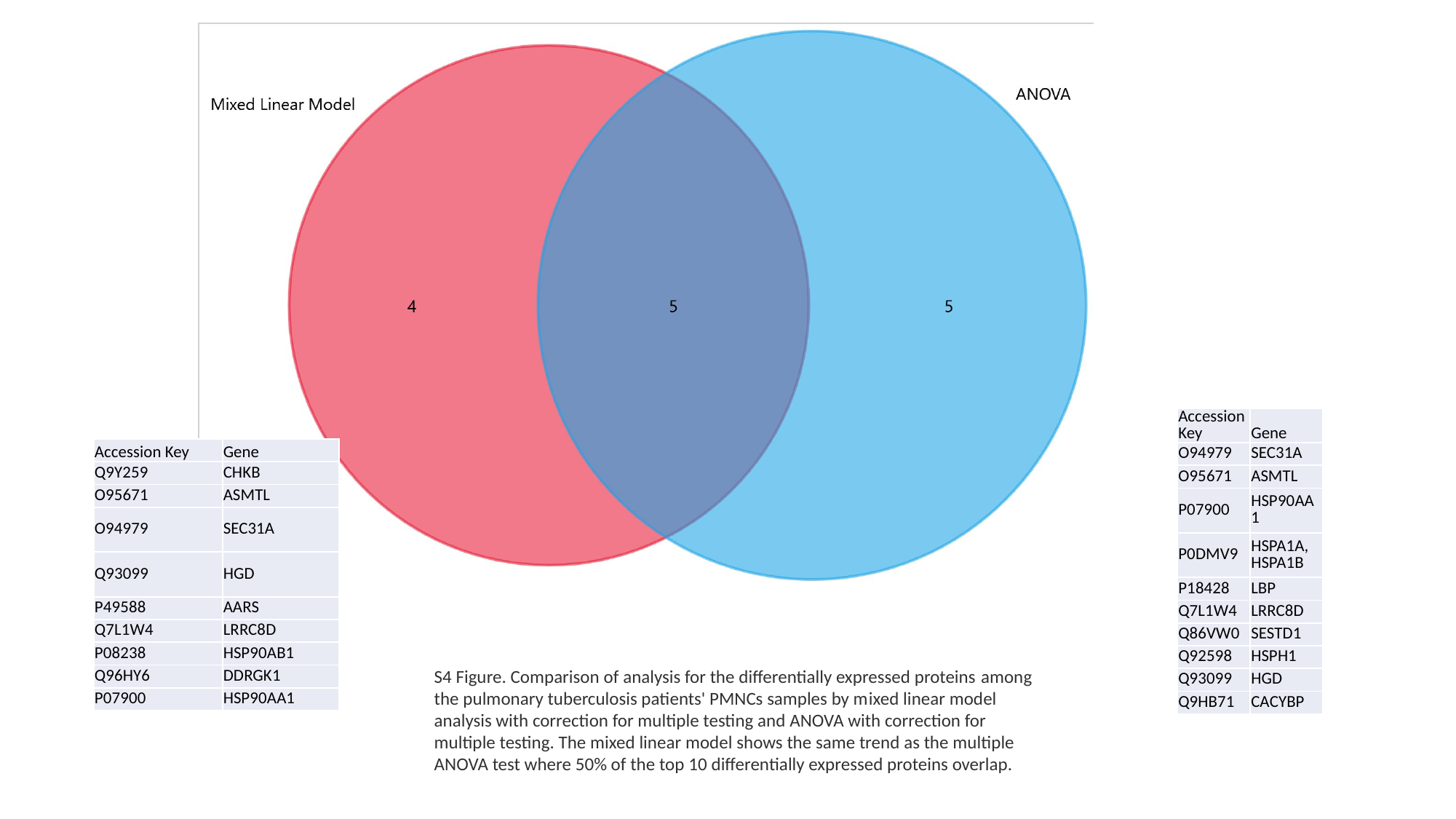

| Accession Key | Gene |
| --- | --- |
| O94979 | SEC31A |
| O95671 | ASMTL |
| P07900 | HSP90AA1 |
| P0DMV9 | HSPA1A, HSPA1B |
| P18428 | LBP |
| Q7L1W4 | LRRC8D |
| Q86VW0 | SESTD1 |
| Q92598 | HSPH1 |
| Q93099 | HGD |
| Q9HB71 | CACYBP |
| Accession Key | Gene |
| --- | --- |
| Q9Y259 | CHKB |
| O95671 | ASMTL |
| O94979 | SEC31A |
| Q93099 | HGD |
| P49588 | AARS |
| Q7L1W4 | LRRC8D |
| P08238 | HSP90AB1 |
| Q96HY6 | DDRGK1 |
| P07900 | HSP90AA1 |
S4 Figure. Comparison of analysis for the differentially expressed proteins among the pulmonary tuberculosis patients' PMNCs samples by mixed linear model analysis with correction for multiple testing and ANOVA with correction for multiple testing. The mixed linear model shows the same trend as the multiple ANOVA test where 50% of the top 10 differentially expressed proteins overlap.

## Slide 5
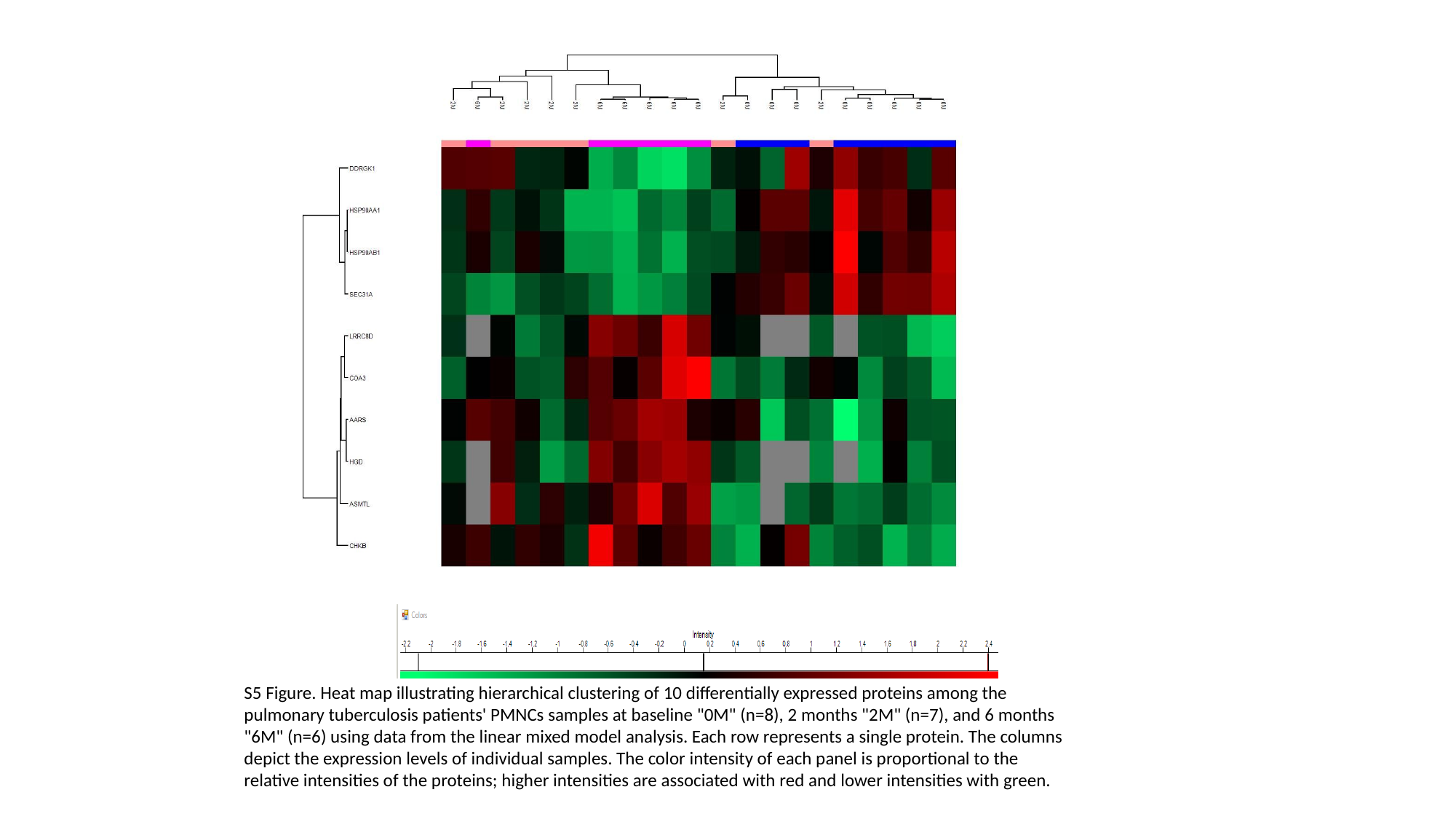

S5 Figure. Heat map illustrating hierarchical clustering of 10 differentially expressed proteins among the pulmonary tuberculosis patients' PMNCs samples at baseline "0M" (n=8), 2 months "2M" (n=7), and 6 months "6M" (n=6) using data from the linear mixed model analysis. Each row represents a single protein. The columns depict the expression levels of individual samples. The color intensity of each panel is proportional to the relative intensities of the proteins; higher intensities are associated with red and lower intensities with green.
